# Whiteboard animation for knowledge mobilization: a test case from the Slave River and Delta, Canada

**DOI:** 10.3402/ijch.v74.28780

**Published:** 2015-10-26

**Authors:** Lori E. A. Bradford, Lalita A. Bharadwaj

**Affiliations:** School of Public Health, University of Saskatchewan, Saskatoon, Canada

**Keywords:** knowledge mobilization, whiteboard animation, traditional knowledge, e-storytelling, Indigenous studies

## Abstract

**Objective:**

To present the co-creation of a whiteboard animation video, an enhanced e-storytelling technique for relaying traditional knowledge interview results as narratives.

**Design:**

We present a design for translating interview results into a script and accompanying series of figures, followed by technical steps to create a whiteboard animation product.

**Method:**

Our project used content analysis and researcher triangulation, followed by a collaborative process to develop an animated video to disseminate research findings. A 13-minute long whiteboard animation video was produced from a research study about changing environments in northern Canadian communities and was distributed to local people. Three challenging issues in the video creation process including communication issues, technical difficulties and contextual debate were resolved among the supporting agencies and researchers.

**Conclusions:**

Dissemination of findings is a crucial step in the research process. Whiteboard animation video products may be a viable and culturally-appropriate form of relaying research results back to Indigenous communities in a storytelling format.

Oral storytelling traditions in Indigenous communities contribute in several distinct ways to the maintenance of traditional knowledge, educational and social development of the people, culture and advocacy for change (1–4). Storytelling, for young children, acts as a precursor to reading and writing across cultures; for older children and youths, storytelling has the additional advantage of solidifying social norms and expectations, teaching moral lessons and reinforcing appropriate behaviours; and for all involved, storytelling benefits the culture evolution and identity formation across social groups (5). Storytelling is socio-culturally significant (6); it provides for intergenerational communication of essential ideas and honours Indigenous people's customs and epistemologies (7). Indigenous communities in Canada continue to raise concern about the erosion of their lifestyles, culture and languages (8–11). Given the difficulties Indigenous people face in maintaining these important aspects of their lives, advocating against and taking action to adapt to confounding environmental changes is a monumental task, let alone gathering and disseminating the data to support their concerns (12–14).

Our research team is concerned with the emerging capacity of Indigenous groups in Canada's North to monitor environmental changes, and most critically, to mobilize research findings in meaningful ways to policymakers and to communities impacted by the changes. In addition to co-creating the *Slave Watershed Environmental Effects Program* (*SWEEP*), a monitoring program, the team sought to devise a tool that advocates for potential and perceived health and environmental issues arising from these changes using culturally relevant means that transcend technological and demographic boundaries. Digital storytelling, primarily by the combination of Photovoice, PowerPoint slides and narration, has been utilized to engage Indigenous communities in climate-health and eco-health research (15,16) and for the purposes of health promotion (17). This paper, however, describes describes a unique digital media design and method that utilizes e-storytelling via whiteboard animation driven by community members and collaborators to relay traditional knowledge gathered through open-ended interviews.

## Research context

Knowledge mobilization includes services that enhance connections between researchers and research users so that research and evidence can inform decisions about public policy and professional practice (18). Improving knowledge mobilization leads to improvements in research uptake, policy design and practice across a variety of sectors and organizations (19,20). As engaged practitioners, we should not be afraid to try new techniques to overcome knowledge mobilization barriers (21–23). There are practical challenges for knowledge mobilization such as turning theory into palatable forms for wider audiences, failing to adopt evidence-based practices, bringing technological advancements to market quickly and overcoming lags between discovery and uptake (19,24,25).

There is also the epistemological challenge of understanding differences between what knowledge “is” in different disciplines, contexts and societies (26,27). An example of where this difference in expression of knowledge and subsequently its mobilization is prevalent is in Canada's North. Traditional knowledge in the North is highly valued and used in policy decision making; however, the extent to which it is passed on and recorded for perpetuity is unknown (28–30). There have been calls from researchers, governments and local people for new, engaging and culturally-appropriate means of mobilizing knowledge of Canada's changing northern regions, whether produced using conventional or co-created means (31–33).

Newly-emerging digital technologies are expanding data-gathering and communication strategies available to health researchers (15–17). In particular, digital storytelling is recognized as an innovative community-based participatory method that increases community members' participation in research on local health issues (34). Digital storytelling, the process of uniting audio, photographs, video and music to create short first-person digital narratives, is an effective way of mobilizing knowledge about climate-health, eco-health and mental health research for popular audiences (15–17). This type of digital storytelling has been demonstrated as a promising and effective engaged research method, capable of creating locally-relevant narratives and culturally-appropriate health communication tools through its decolonizing process (15,35). Digital storytelling benefits primary education initiatives through increasing student access to alternative forms of literacy (36,37). It is also recognized as a means for sharing Indigenous empirical material in research outputs (17).

In short, arts-based health research initiatives such as digital storytelling have emerged as a fruitful avenue for innovation in qualitative research, for developing culturally-appropriate health communication platforms, and as a means for disseminating research results (38). Few examples of the use of digital storytelling in the northern Indigenous context have been provided (15,17). To that end, this paper describes the protocol and application of an enhanced form of digital storytelling for sharing traditional knowledge produced through open-ended interviews for knowledge holders, youth and others in a northern Indigenous context.

## Site context and design

Communities in the Northwest Territories (NWT) are concerned about the health of the Slave River and Delta (SRD). Industrial activities upstream in the Peace-Athabasca and Slave River watersheds are impacting water quantity and quality in the SRD. In response to community concerns about the health of the ecosystem and to facilitate community-based monitoring in the SRD, the Slave River and Delta Partnership (SRDP) was created in 2010 by the Government of the NWT Environment and Natural Resources (GNWT-ENR) Lands and Water Division, as part of the NWT Water Stewardship Strategy. The SRDP is a collaboration of agencies and organizations working and living in the SRD basin. Partners include 5 First Nations and Métis organizations, 1 territorial government agency, 4 federal government agencies, and Aurora College and Aurora Research Institute. This project design is one part of an ongoing research program working collaboratively with the SRDP seeking to identify, prioritize, measure and report on indicators of environmental health in the SDR communities. The project received research ethics and community approval (University of Saskatchewan REB 13-165 and Aurora Research Institute License No. 15383).

The digital storytelling and whiteboard animation design emerged from stakeholder feedback indicating a desire to have results from traditional knowledge interviews relayed back to the community in a non-textual format, and a format that would endear youths in the community. We began with interviewing 11 Elders and local people for their ethnographical accounts of the changes in the river and delta across their lifetimes (see Appendix 1 for interview guide). Topics related to the conditions of the SRD, any observed changes to wildlife, aquatic species, plants and water from past to present and future were discussed. Interviews were audio-recorded in English and transcribed. The researchers analysed the transcripts and came to consensus on 5 main themes that captured the content of the interviews. The themes became the focus for the whiteboard animation table of symbolic images (Table I). The focus of the video was threefold; to give voice to the Elders in a storytelling format that transcends the cultural inappropriateness and fading era of the printed page (39), to narrate multiple perspectives of ethnographic history of the region undergoing rapid and extensive environmental change, and to visualize the interview content as a single story that can be broadcast more widely and in an e-format.

**Table I:** Research translation from interview transcripts to symbolic images

Interview themes		Representative symbols and descriptions
Where We Came From		
	Places where participants were born	Indicated as dots on an overall map of the region drawn with rivers and delta channels shown
	Stories of activities when they were young	Fishing: 2 boys silhouettes with footprints walking away holding fishing poles
		Hunting and skinning hides: moose, bear, skinning knife
	Historic towns and settlements	Family drawn in front of teepee, church building drawn in background, flames drawn on it to indicate it burned down
	Growing up on the land	Hut in the woods, hides drying on lines outside hut, stars in the night sky
	Change in the movement of people	Compass symbol, map of range of travel of South Slave people, sunset and horizon
	Herding caribou	Men, women and children drawn herding caribou
	Decades where lifestyles began changing	Decades written in numbers, then arrow drawn to boat with child and grandparent, a mittened hand holding up a muskrat, flock of geese flying, then an arrow drawn to locations on previous map, and people gathered drinking tea
Development and Disease		
	Railway, mines and abandonment of earlier settlements and nomadic lifestyle	Railway tracks, a cross to indicate a mission in a community, supply barge drawn then erased, open pit mine layers, dump truck outlines
	Northern dam and reservoir filling	Cross-section of dam drawn, then water level increased behind it, a trickle of water drawn down other side, canoe drawn beside small stream
	Changing transportation	Dogsled drawn, then erased and a snowmobile drawn instead, then a road, and a line of transport trucks
	Pollution and diseases	Dirt drawn in air and landing on snow, caribou drawn eating shoots coming up through pollution on snow, large tick drawn, grocery cart to indicate changing food patterns in the North
Our Food Is Changing		
	Listing of traditional foods	Medicine wheel is drawn with images of symbolic animals at compass points (i.e. duck, hare, fish, moose, bear, muskoxen)
	Perceived toxicity of traditional foods	Images of animals are faded in, then erased as narrator dictates the perceived problems with traditional foods (i.e. “You might shoot a moose, but find it's sick or infested, you just leave it.”)
	Bird population is changing	Erase parts of flocks drawn earlier, fade shoreline in and out
	Food web is changing	Erase some of the channels from the previous map
Our Water Is Changing		
	The ice isn't solid or predictable anymore	People skating on the river, then big cracks appear, a thermometer is drawn and circled, calendars are drawn and a big red X is marked after 3 calendar pages are checked off, a man with an auger drills core samples and looks confused
	Ice changes are affecting traditional food sources and wildlife	A beaver is drawn under the ice, frozen while trying to chew its way out, a man is drawn looking down through the ice at the beaver
	The water level and dynamics of the river have changed	The water by the cabin's edge is drawn approaching the cabin with the level going up and the colour changing as in a flood, the flooding is then erased. The artist returns to the map and draws a circle around it depicting a cycle, then erases channels and adds channels to show the cycle. A sponge is drawn and water floods around it. Plants appear growing around the sponge. Circles are again drawn around the map.
The Balance Is Off		
	The interconnectedness of water, the delta, and the spiritual understanding of the people	Water droplets are drawn below the medicine wheel from earlier. Each water droplet is a deepening level of a cosmological understanding with 4 parts; for example, the first droplet discusses how individuals are made up of hearts, bodies, minds, and spirits. Seven droplets representing 7 levels of spirituality and connectedness are drawn smaller as they drip down towards a bucket.
	The balance is thrown off by changing water flow and use in the region	Labels from within the droplets are erased sequentially illustrating how changes to one level of spirituality affect the others levels. A shoe is drawn kicking over the bucket of water.
	The people want the water cycle to be returned	Water is drawn spilling from the bucket and channelling into a river. The river and delta is redrawn. Channels are added and erased. The video then pans out to the entire whiteboard drawing as the closing lines are read.

## Method

The creation of the video project was a single, exploratory test of a method to disseminate interview findings in a digital storytelling format. In the SWEEP, we accepted our role as “marginal” tools, under the direction of community members, but the video was not decolonizing, or participatory action research (40). The larger SWEEP program for environmental monitoring was co-designed with community members; involved western science and traditional knowledge indicator measurements; and had a vision of deriving a monitoring program built and scaffolded with university-based researchers in the first 2 years, then wholly owned and actioned by local community members for as long as practicable. Interviews were guided by a pragmatic western social science perspective; that is, we used a series of questions and answers between 2 people to gather information (41). For the video we, the authors, analysed the transcripts; developed the table of symbolic images; and sought feedback from the community members, scientists and other partners on our interpretations.

The 11 interviews (9 male, 2 female; age range: 47–80 years, average age: 67) took place between April and November 2014 in 2 locations. The first location was Fort Smith, NWT (a tourism, government and education-based town of about 2,500 people with an ongoing strong traditional economy based in hunting and the fur-trade) and, the second location was Fort Resolution, NWT (a hamlet that grew from a mining and fur-trading background, currently based in traditional economies). The interviews were audio-recorded with permission and ranged from 45 minutes to over 3 hours. Participants were shown several pictures and maps of the SRD region ranging from the 1940s up to 2015. Participants could point out places on the map that were important to them while they were telling their stories, if desired.

The interview transcripts underwent content analysis (42), and emerging patterns and insights were categorized alongside a listing of special events, places and activities that occurred as a part of daily life in the region. The categories were broadly based on the faces of traditional ecological knowledge (43); there were observations of environmental changes; changes to harvesting and use of resources (management); and changes to identity, culture and cosmology. The findings were then sequenced chronologically with input from the participants through follow-up e-mail and telephone conversations. Many participants relayed the dates and locations of certain key events during the development of the region, which aided in this process.

A single, encompassing narrative account of a “lifetime” in the region was developed using data from all 11 interviews across 5 broad themes: where the participants came from (early life experiences), development and disease, changing foods, changing waters and changing spiritual understandings of the world. The narrative account was massaged into a script. At times, the script contained quoted text from the interviews with participant permission. A sequence of images was then created alongside the script. Symbols were used to represent activities, places or events in the history of the region; for example, 2 sets of footprints leading to silhouettes of 2 boys carrying fishing rods was used to depict a narrative sequence where a participant remembered going fishing with his brothers as a young boy; a sketch of the outline of a dam and sequence of the reservoir behind filling with water over time was used to represent the construction and filling of the W. C. Bennett Dam and Williston Reservoir upstream in British Columbia, during the late 1960s and 1970s; and a sequence of falling water droplets containing medicine wheel elements was used to depict a traditional knowledge system of the region's connection to the waters of the river and delta (Table I).

## Technical details

**Fig. 1:**
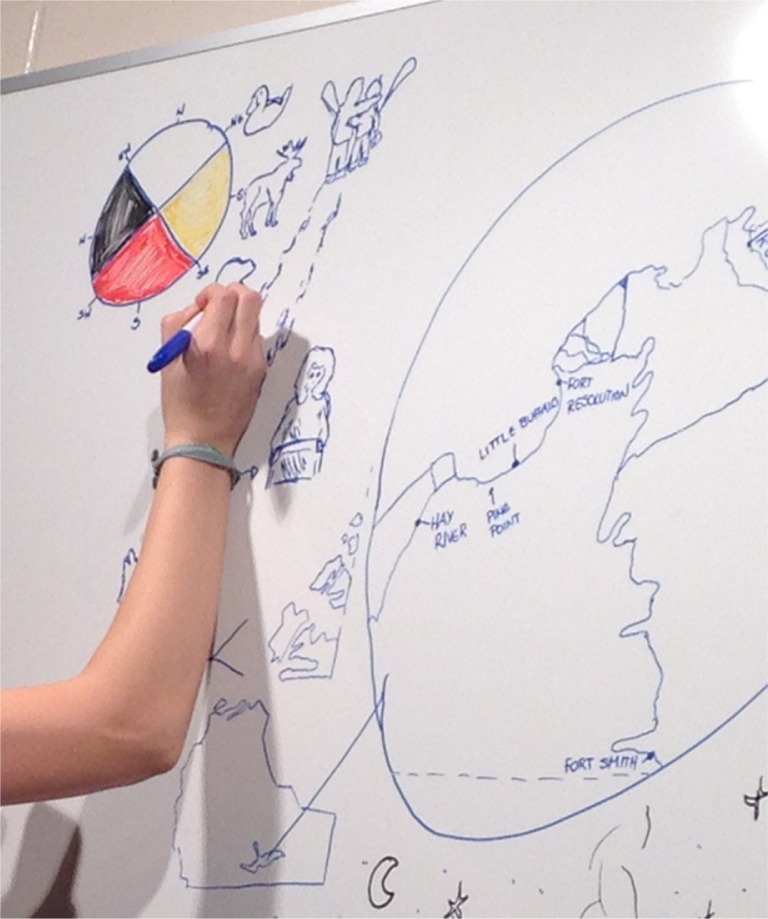
Drawing on the whiteboard.

A high-quality camera capturing 5 images a second was set up in front of a large whiteboard. A fourth-year Fine Arts student with Indigenous ancestry was recruited to draw key images on the whiteboard. The student prepared by examining different images online, consulting with relatives and creating a set of drawings on paper to scaffold the animation sequencing. Images drawn to visually depict the verbal descriptions within the stories were shared with our Indigenous partners to confirm the accuracy of the drawings, for example, the illustration of a trapper's cabin or the flooding of the delta. Once finalized, the student carried out the drawing on the large whiteboard in 2 separate sequences as it was a lengthy and physically challenging process of drawing with her arm held up (Fig. 1). The camera took images of the entire whiteboard at high resolution during the drawing. An upper-year Fine Arts (Drama) student with Indigenous ancestry performed the narration separately from the illustration. The recording and animation came together in a later step in the project. During this time, researchers also gathered suggestions for music from people living in the SRD region, and selected a piece of music performed by a band with members coming from the region.

The technical team at the university's media and production unit zoomed the images to focus on the artist's hand and forearm as she drew the pictures. Once the zoom level and sequencing were established, camera steadiness was resolved and the 8 hour long sequence of images was sped up to look like it was a continuous 13-minute video of an arm drawing the chosen images. The narration was edited to fit the timing of particular images, and music and soundscaping were added. Some special effects were used to ensure the sequence and timing were visually appealing.

Draft segments from the video were shared with Indigenous partners and individual research scientists involved in the larger program to confirm the accuracy of the narrative. Corrections to species names, locations and ice dynamics were made as a result. These segments were also shown to supporting agencies for feedback during several conference calls. Editing was completed at the request of partners and the final whiteboard animation video was gifted to the community during two showings in May 2015. Community members decided to allow broadcast of the video more widely (i.e. through social media) after an arranged public viewing. The video can be viewed at www.youtube.com/watch?v=XHjmcdNwVpE

## Overcoming challenges

The process of completing the video was not tension-free. We faced 3 main challenges: there were communication difficulties, technical issues to overcome and contextual debate (see below).

Communication difficulties arose when working with interview participants to finalize symbols and ensure accuracy. It was difficult for interview participants to understand how the images were going to represent parts of the story without the full animation sequence with the narrative included to show them. Doing so would have required more time and resources in the production stage. Participants also lived remotely and were engaged in traditional livelihoods during the production stage, thus not always able to verify that edits captured their desired changes. Culturally-important symbols such as the medicine wheel needed to be included in sensitive and appropriate ways, but while government agency representatives were cautious about using the medicine wheel, when asked, interviewees and community members welcomed the familiarity of the graphic. The accuracy of maps was also important to the community members who contributed hand-drawn pictures of what the channels looked like, as well as historic photographs they owned. Time pressures and a shortage of funding were also of concern. While the artist needed time and space to draw freely and creatively, and she had prepared by sharing various ideas with the researchers and technical team, the actual capturing of the drawing was a time-intensive part of the process. These constraints made deciding on the content of the final video challenging. We found that using the table to build understanding of the compiled storyline was helpful. To better communicate the inception and goal of the video, researchers and funders suggested including an introduction and conclusion that depicted the larger research program and ongoing efforts.

Technical issues were also faced; because of the need for the still images to be captured at a high rate to make the animation sequence look realistic, and because of memory limitations of devices, the actual drawing was captured in segments that needed to be merged in the final product. The camera “bounce” due to the frequency of picture taking also needed repairing which was completed with “coding” designed by the technical producer. Photographic images shared by research team members from the larger research program were included in the introduction and closing of the video which meant extra production time was necessary.

The third challenge involved contextual debate. The researchers at times felt tension between including the messaging from supporting government agencies and research centres, and the themes that emerged from the participants’ interviews. One particularly contentious issue arose during the sharing of draft segments of the video. Government agency partners flagged a segment depicting perceptions of safety and contamination of country foods. The sequence used the medicine wheel to show the relationships between certain harvested food items and importance to traditional lifestyles. The sequence included the erasing of animals as their perceived toxicity is described (i.e. “We can't eat bear anymore, they eat from the dump.”), while an image of a skull and crossbones is drawn inside the medicine wheel. The picture is controversial because the messaging from the Government of NWT and the perception of the local people is that country foods are on the whole, healthier choices than unhealthy choices in the grocery stores or community food programs (see, for example, (44–46). Seven of the 11 interview transcripts, however, contained the words toxic, poison, “no good,” or “waste” when referring to the country foods drawn around the medicine wheel. After deliberation, the image of the skull and crossbones was removed from the video, and other images of country foods discussed in the narration were added in its place.

## Discussion

Research translation through whiteboard animation has potential for complementing conventional knowledge mobilization outputs such as presentations, conference proceedings and manuscripts. A formal evaluation of the final whiteboard animation video was not conducted; however, written notes and expressions were recorded following the viewings with our Indigenous partners. Our partners commented that the video was emotionally appealing and reflective of their lived experiences. They also stated the video could act as an engaging introduction for workshops on environmental and cultural changes in the region so that those people in attendance less familiar about the region's history could quickly learn about the perceived sources of impacts to livelihoods, and how these impacts are affecting the worldviews of the local people. This supports other work indicating that arts-based products can be successful at situating results within wider community concerns (21,22), and work to broker difficult discussions among diverse groups (23,38).

Community members indicated that our sample and, subsequently, our narrative was skewed towards people who dwelled in the delta and less about life in upstream river locations. As a result, much of the video's ecosystem knowledge focused on delta dynamics and wildlife with few relevant upstream examples. Feedback such as that provide to us through the viewing of this video helps us recognize our deficiencies, and reiterating why, as researchers knowing, (i.e. gathering data and analysing it), is not enough (20). Within the larger program, we hope to contribute future knowledge mobilization products to balance the downstream knowledge creation bias.

Our product differs from other knowledge mobilization digital storytelling products in three key ways; first, we created the video with Indigenous participants providing feedback during the production stages. Research scientists, representatives from supporting agencies and funding partners contributed to the reviewing of draft videos. Second, the narrative was a compilation of 11 different interview participants’ stories and traditional knowledge. Where many digital storytelling outputs are short pieces including individual perspectives on a topic (e.g. 15–17), and photographic libraries, our video tells the story of Indigenous groups struggling against dramatic environmental and social changes in their region. We also used symbolic hand-drawn images which were “animated” through digital technology to tell the story. The inclusion of university students in our practice meant the knowledge was mobilized further afield through their networks, and their own portfolios were bolstered.

Agencies seeking to co-produce knowledge with communities and researchers could benefit from these types of projects because of the ability to condense large amounts of data into accessible formats for northern communities and Indigenous people. Our First Nations and Métis partners indicated they enjoyed the video and validated its local relevance and ability to reach people. They believed it would be an effective educational tool for use in schools to identify culturally-important historical events, and notable places in their region. Copies of the video have been placed in the local schools for use as an educational tool and it is streaming live in the Health Clinic of one of our partnering communities.

There are important lessons learned from the transdisciplinary collaboration in this project. Our research design and technical production needed to allow for more consultation with both the supporting agencies, and the local people so that the balance between advocacy and information sharing could be established early on. The debates that emerged could have been eased with earlier agreement on particular themes emerging from the interviews. We also needed to ensure that everyone agreed on the list of images to be drawn for the artist so that there would be less post-production editing.

## Conclusions

Arts-based approaches make visible peoples’ experiences that are often left unarticulated or hidden (26). This project sought to overcome some barriers of knowledge mobilization specific to the northern Canadian Indigenous context. Creative e-based dissemination approaches such as whiteboard animation can promote insight and new ways of co-producing knowledge that communicates traditional knowledge to wider audiences.

## Appendix 1

Interview Guide: SRD Traditional Knowledge Program

1. Tell us about yourself (i.e. when/where you were born, your family, your home).
2. Please share with me how you remember the SRD back in your childhood, youth, young adult years (in the 1940's, 50's, 60's, 70's, 80's, …). Please tell us what the:
  - Water was like?
  - Delta was like?
  - Plants, fish, birds and animals that were around?
  - Ice conditions were like?
3. Please share how you remember people living on and within the SRD region.
  - Hunting and trapping?
  - Gathering food, plants, water, medicines, other resources?
  - Getting around?
  - Community events and ethics?
4. In your view, what events, activities, projects have affected the SRD?
  - As a result of these events what changes have you seen in the river/delta?
  - Tell us how you know these changes happened?
  - How have the changes personally affected you?
5. Can you share with me any changes in the delta that have been shared with you from your parents (Elders and grandparents)?
6. What do you think the SRD will be like in the future?
7. What stories do you want to share about the special places, parts and people of the SRD?

## References

[1] Battiste M (2002). Indigenous knowledge and pedagogy in First Nations education: a literature review with recommendations.

[2] Piquemal N (2003). From Native North American oral traditions to Western literacy: storytelling in education. Alberta J Educ Res.

[3] Dowell K (2006). Indigenous media gone global: strengthening Indigenous identity on-and offscreen at the First Nations/First features film showcase. Am Anthropol.

[4] McCall S (2011). First person plural: Aboriginal storytelling and the ethics of collaborative authorship.

[5] McKeough A, Bird S, Tourigny E, Romaine A, Graham S, Ottmann J (2008). Storytelling as a foundation to literacy development for Aboriginal children: Culturally and developmentally appropriate practices. Can Psychol.

[6] Dei GJ (2000). Rethinking the role of indigenous knowledges in the academy. Int J Incl Educ.

[7] Iseke-Barnes J (2003). Living and writing indigenous spiritual resistance. J Intercul Stud.

[8] Canadian Heritage (2005). Towards a new beginning: a foundation report for a strategy to revitalize First Nation, Inuit and Métis Languages and Cultures. Report to the Minister of Canadian Heritage by The Task Force on Aboriginal Languages and Cultures.

[9] Norris MJ (2007). Aboriginal languages in Canada: emerging trends and perspectives on second language acquisition. Can Soc Trends.

[10] Angell AC, Parkins JR (2011). Resource development and aboriginal culture in the Canadian north. Polar Rec.

[11] Battiste M, Burke SZ, Milewski P (2012). Enabling the autumn seed: toward a decolonized approach to Aboriginal knowledge, language, and education. Schooling in transition: readings in Canadian History of Education.

[12] Berkes F, Mathias J, Kislalioglu M, Fast H (2001). The Canadian Arctic and the Oceans Act: the development of participatory environmental research and management. Ocean Coast Manag.

[13] O'Faircheallaigh C (2007). Environmental agreements, EIA follow-up and aboriginal participation in environmental management: the Canadian experience. Environ Impact Assess Rev.

[14] Parlee BL, Goddard E, First Nation ŁKÉD, Smith M (2014). Tracking change: traditional knowledge and monitoring of wildlife health in Northern Canada. Hum Dimens Wildli.

[15] Cunsolo Willox A, Harper S, Edge V (2013). The ‘My Word’ Lab, and the Rigolet Inuit Community Government. Storytelling in a digital age: digital storytelling as an emerging narrative method for preserving and promoting Indigenous oral wisdom. Qual Res.

[16] Parkes MW, Bowering D (2013). “Upstream is a place”: learning together about watersheds as intersectoral settings for health.

[17] Gubrium A (2009). Digitial storytelling: an emergent method for health promotion research and practice. Health Prom Prac.

[18] Phipps DJ, Shapson S (2009). Knowledge mobilisation builds local research collaborations for social innovation. Evid Policy.

[19] Levin B (2008). Thinking about knowledge mobilization. In an invitational symposium sponsored by the Canadian Council on Learning and the Social Sciences and Humanities Research Council of Canada.

[20] Levin B (2013). To know is not enough: research knowledge and its use. Rev Educ.

[21] Rossiter K, Gray J, Kontos P, Keightley M, Colantonio A, Gilbert J (2008). From page to stage: dramaturgy and the art of interdisciplinary translation. J Health Psychol.

[22] Rossiter K, Kontos P, Colantonio A, Gilbert J, Gray J, Keightley M (2008). Staging data: theatre as a tool for analysis and knowledge transfer in health research. Soc Sci Med.

[23] 
Phipps D, Morton S (2013). Qualities of knowledge brokers: reflections from practice. Evid Policy.

[24] McDermott R, O'Dell C (2001). Overcoming cultural barriers to sharing knowledge. J Know Manag.

[25] Riege A (2005). Three-dozen knowledge-sharing barriers managers must consider. J Know Manag.

[26] Dilling L, Lemos MC (2011). Creating usable science: opportunities and constraints for climate knowledge use and their implications for science policy. Global Environ Change.

[27] Bruce A, Makaroff KLS, Sheilds L, Beuthin R, Molzahn A, Shermak S (2013). Lessons learned about art-based approaches for disseminating knowledge. Nurse Res.

[28] Ellis SC (2005). Meaningful consideration? A review of traditional knowledge in environmental decision making. Arctic.

[29] Pearce TD, Ford JD, Laidler GJ, Smit B, Duerden F, Allarut M (2009). Community collaboration and climate change research in the Canadian Arctic. Pol Res.

[30] Cameron ES (2012). Securing Indigenous politics: a critique of the vulnerability and adaptation approach to the human dimensions of climate change in the Canadian Arctic. Glob Environ Change.

[31] Parlee B, Manseau M (2005). Using traditional knowledge to adapt to ecological change: Denésǫłıné monitoring of Caribou movements. Arctic.

[32] Gearheard S, Shirley J (2007). Challenges in community-research relationships: learning from natural science in Nunavut. Arctic.

[33] Brunet ND, Hickey GM, Humphries MM (2014). The evolution of local participation and the mode of knowledge production in Arctic research. Ecol Soc.

[34] Lambert J (2007). Digital storytelling capturing lives, creating community.

[35] Willox AC, Harper SL, Edge VL (2012). Storytelling in a digital age: digital storytelling as an emerging narrative method for preserving and promoting indigenous oral wisdom. Qual Res.

[36] Educause Learning Initiative (2007). 7 things you should know about … digital storytelling.

[37] Ohler JB (2007). Digital storytelling in the classroom: new media pathways to literacy, learning and creativity.

[38] Boydell KM, Gladstone BM, Volpe T, Allemang B, Stasiulis E (2012). The production and dissemination of knowledge: a scoping review of arts-based health research. Forum Qual Soc Res.

[39] Cherubini L (2008). The metamorphosis of an oral tradition: dissonance in the digital stories of Aboriginal peoples in Canada. Oral Trad.

[40] Zavala M (2013). What do we mean by decolonizing research strategies? Lessons from decolonizing, indigenous research projects in New Zealand and Latin America. Decolon: Ind Ed Soc.

[41] Ormston R, Spencer L, Barnard M, Snape D, Ritchie J, Lewis J, Nicholls CM, Ormston R (2013). The foundations of qualitative research. Qualitative research practice: a guide for social science students and researchers.

[42] Patton MQ (1980). Qualitative evaluation methods.

[43] Houde N (2007). The six faces of traditional ecological knowledge: challenges and opportunities for Canadian co-management arrangements. Ecol Soc.

[44] Ford JD, Lardeau MP, Blackett H, Chatwood S, Kurszewski D (2013). Community food program use in Inuvik, Northwest Territories. BMC Publ Health.

[45] Muir D, Kurt-Karakus P, Stow J, Northern Contaminants Program (NCP) (2013). Canadian Arctic contaminants assessment report on persistent organic pollutants – 2013. http://www.science.gc.ca/default.asp?lang=En&n=6D4B6162-1.

[46] Seabert TA, Pal S, Pinet B, Haman M, Robidoux MA, Imbeault P (2014). Elevated contaminants contrasted with potential benefits of ω-3 fatty acids in wild food consumers of two remote First Nations Communities in Northern Ontario, Canada. PLoS One.

